# COVID-19 Recovery and Cardiovascular Health: The Interplay Between Fetuin-A and Blood Pressure

**DOI:** 10.7759/cureus.69905

**Published:** 2024-09-22

**Authors:** Montather F Ramadan, Iqbal J Al-Assadi, Foued B Hadj Slama

**Affiliations:** 1 College of Dentistry, Al-Ayen University, Thi-Qar, IRQ; 2 Faculty of Medicine Ibn El Jazzar, University of Sousse, Sousse, TUN; 3 Department of Chemistry, College of Science, Basra University, Basra, IRQ

**Keywords:** blood pressure, cardiology, cardiovascular health, covid-19, fetuin-a

## Abstract

Introduction: COVID-19 has been shown to impair cardiovascular function, and further studies have proven that this effect can be long-term on several cardiovascular biomarkers. Fetuin-A, a multifunctional protein involved in calcification and inflammation, has emerged as an important biomarker in this process. This study investigates the relationship between recovery from COVID-19, cardiovascular health, and concentrations of fetuin-A in patients with high blood pressure.

Methods: Seventy-nine men and 36 women were admitted to the Nasiriyah Heart Center in Iraq between March and August 2023, with ages ranging from 5 to 93 years. Clinical data were collected on admission along with blood samples, and serum levels of fetuin-A were measured using an enzyme-linked immunosorbent assay (ELISA). The results were analyzed using Python libraries Pandas and SciPy to perform independent sample t-tests to determine mean levels of fetuin-A in various patient subgroups. A p-value of less than 0.05 was considered statistically significant.

Results: The study showed that patients who had survived COVID-19 had significantly higher levels of fetuin-A compared to healthy controls, with a mean concentration of 103.64 mg/L versus 19.199 mg/L (p < 0.001). Additionally, it was found that patients with high blood pressure had increased levels of fetuin-A compared to those without high blood pressure, with a mean concentration of 109.01 mg/L versus 95.88 mg/L (p = 0.025). These results suggest that COVID-19 may alter the usual relationship between blood pressure and cardiovascular biomarkers.

Conclusion: This study emphasizes the complex interaction between recovery from COVID-19 and cardiovascular health, primarily through the levels of fetuin-A. The increase in fetuin-A among hypertensive patients suggests that COVID-19 may enhance cardiovascular risk, highlighting the need for stricter monitoring and tailored treatment strategies. Further studies are needed to elucidate the underlying mechanisms, which will help develop effective clinical guidelines for managing cardiovascular health in COVID-19 survivors.

## Introduction

Overview of COVID-19 background

COVID-19 is a contagious respiratory disease that began in December 2019 in Wuhan, China. It is caused by a new virus, now known as SARS-CoV-2, which belongs to the family of coronaviruses. Coronaviruses are in the family of human airborne disease viruses and cause about one out of every four cases of mild illness during the winter season, with the most common symptoms being fever, cough, and breathing difficulties. However, this outbreak is due to a new strain that had not been seen before in humans. This new strain has led to a global outbreak, affecting millions of people worldwide [1,2]. It reached many countries and was extremely severe in its effects, causing many illnesses, putting tremendous pressure on healthcare systems, and having significant economic impacts. This has led to measures like social distancing, increased testing, and vaccine development by governments and healthcare facilities. These efforts have seen some success in reducing transmission rates and new cases, but the fight against COVID-19 is ongoing, especially with new variants of the virus emerging, which have made containment and prevention more difficult. The scientific community is focused on understanding the virus's behavior to develop better treatments and preventive measures. However, this does not eliminate the need to follow public health protocols to reduce the risk of infection or transmission. Proper hygiene, mask-wearing, and vaccination can help the public contain the spread of COVID-19 and, in the long run, assist in stopping this pandemic [3,4].

Atypical presentations and long-term effects

During the course of the COVID-19 pandemic, it has been learned that the disease can produce a wide spectrum of manifestations and clinical profiles. Atypical symptoms, which do not have the characteristic features of COVID-19, have been reported and described more frequently [5-8]. These atypical manifestations include neurological, gastrointestinal, and/or cardiovascular symptoms [5,9]. Managing cases with unusual presentations has posed significant challenges since such symptoms may not initially appear related to COVID-19 [7,8]. However, as the pandemic has progressed, many of these less typical symptoms have become better understood and more widely recognized within the medical community [7]. Nevertheless, some rare and atypical manifestations still present challenges in terms of detection and diagnosis [5]. Beyond the typical severe manifestations, long-COVID has also emerged as a significant issue. Some of the most common symptoms from long-COVID include fatigue, dyspnea, and arthralgia [7,10]. The pathophysiological processes and causes of these late sequelae are still not fully understood, and more research is needed to determine the interaction between the immune and inflammatory systems [5]. Some proposed theories include the existence of chronic viral reservoirs, atypical immune responses, reactivation of other viruses, and widespread systemic symptoms [10]. Identifying those most at risk of developing long-COVID can help better direct prevention and treatment measures [7]. It is imperative for healthcare providers to continue screening and addressing a broad range of symptoms, particularly those affecting cardiovascular health, from the initial onset of illness to post-acute sequelae.

Fetuin-A

Fetuin-A, also referred to as alpha-2-HS-glycoprotein, is a multifunctional protein synthesized mainly in the liver and secreted into circulation, where it plays a significant role in various physiological processes [11]. The primary function of fetuin-A is as a negative regulator of calcium, inhibiting ectopic calcification, the process by which calcium phosphate crystals form in soft tissues and blood vessels. As a result, fetuin-A helps prevent vascular calcification, offering protection against diseases associated with calcification, including cardiovascular diseases. However, while a deficiency of fetuin-A increases the risk of cardiovascular mortality in dialysis patients, elevated levels of this protein are also detrimental to health, increasing the risk of cardiovascular diseases, type 2 diabetes, and metabolic syndrome [11-13].

This dichotomy in fetuin-A’s effects can be understood by examining its diverse functions. Beyond acting as a calcification inhibitor, fetuin-A regulates calcium metabolism, osteogenesis, and insulin signaling by inhibiting the insulin-stimulated autophosphorylation of the insulin receptor. This may explain its association with metabolic disorders such as type 2 diabetes and metabolic syndrome [13]. Additionally, fetuin-A reduces calcium phosphate crystallization in soft tissues and blood vessels, preventing diseases caused by vascular calcification [11,12]. Interestingly, both low and high levels of fetuin-A are linked to health risks: low levels are associated with increased cardiovascular mortality in dialysis patients, while elevated levels are linked to greater susceptibility to cardiovascular diseases, type 2 diabetes, and metabolic syndrome [12,13]. This apparent contradiction reflects the wide range of functions performed by fetuin-A, including calcium homeostasis, bone remodeling, and insulin signaling [14,15].

Furthermore, fetuin-A exhibits both anti-inflammatory and pro-inflammatory effects depending on the type and intensity of the inflammatory response, positioning it as an acute-phase protein [12]. The interaction between fetuin-A and various physiological and pathophysiological processes suggests the need for new approaches to evaluate its role in overall health and disease [11,16].

Relationship with cardiovascular disease

The association between fetuin-A and cardiovascular disease has garnered significant interest. Studies have indicated a direct correlation between low plasma fetuin-A concentrations and an increased occurrence of cardiovascular events, including atherosclerosis, coronary artery disease, and myocardial infarction. Fetuin-A has been identified as a potent calcification inhibitor in blood vessels, a vital process in the development of cardiovascular diseases. Additionally, it impairs insulin signaling, inflammation, and endothelial function, all of which contribute to the development of cardiovascular diseases (CVDs). Studying the exact pathways of the association between fetuin-A and cardiovascular disease may provide some therapeutic strategies for avoiding and managing this common health problem [17,18].

Fetuin-A plays a critical role in maintaining calcium homeostasis and preventing abnormal calcium deposition, which is central to cardiovascular diseases. It is important to note that low levels of circulating fetuin-A have been associated with cardiovascular events such as atherosclerosis, coronary artery disease, and myocardial infarction [11-13].

However, the relationship between serum fetuin-A and cardiovascular events is not entirely clear. An inverse correlation has been observed between the two variables, suggesting that as one increases, the other decreases. On the one hand, a reduction in circulating fetuin-A is associated with an increased risk of arteriosclerosis and other cardiovascular complications such as coronary artery disease and myocardial infarction. This is because fetuin-A acts as a calcification inhibitor in blood vessels, which is essential for the prevention of cardiovascular diseases. As an anti-vascular calcification molecule, fetuin-A contributes to the elasticity and efficiency of blood vessels, thus reducing susceptibility to cardiovascular incidents. Consequently, low levels of fetuin-A are associated with higher cardiovascular mortality, particularly in dialysis patients who are vulnerable to vascular calcification [12,11].

On the other hand, increased plasma levels of fetuin-A have also been linked to an elevated risk of cardiovascular diseases [12,13,19]. This may seem contradictory given fetuin-A's diverse roles in the human body. As an acute-phase protein, fetuin-A can be upregulated in response to various inflammatory mediators and tissue injuries [13].

Elevated fetuin-A levels have been found to correlate with an increased risk of cardiovascular events such as myocardial infarction and stroke. This relationship may be partly due to fetuin-A's role in modulating inflammation and calcification processes within the vascular system. High levels of fetuin-A may contribute to vascular calcification and endothelial dysfunction, which are critical factors in the development of atherosclerosis. A recent study demonstrated that high fetuin-A levels are associated with increased arterial stiffness, a precursor to cardiovascular events [20,21].

Relationship with COVID-19

The involvement of fetuin-A, particularly in relation to COVID-19, is an area of current research interest. Recent findings suggest that elevated concentrations of fetuin-A may be linked to poor prognoses among COVID-19 patients. As a result, recommendations have been made that fetuin-A could serve as a potential biomarker for disease severity and prognosis in COVID-19 cases. Additionally, scientists are exploring how fetuin-A may be involved in the hyperinflammation and endothelial damage commonly seen in severe forms of the disease. Research connecting fetuin-A as a biomarker to COVID-19 could not only assist in predicting disease severity but may also provide insights into how bioactive molecules can be utilized to mitigate the virus's impact [22].

Fetuin-A and blood pressure-related changes

It was also demonstrated that the level of fetuin-A decreases with the progression of arteriosclerosis and the onset of vascular calcification, which are factors contributing to the development of hypertension. Fetuin-A is the inhibitory protein involved in systemic calcification, and its absence may result in calcification of vascular tissues, leading to increased arterial stiffness and blood pressure [23,24].

For instance, one published study showed that aortic arch calcification frequency was higher in patients with low serum fetuin-A levels, which is associated with hypertension. These inverse relationships suggest that fetuin-A has a protective effect against hypertension, mediated by the inhibition of vascular calcification [23]. Fetuin-A inhibits inflammation and could help alleviate the inflammation that worsens in hypertensive patients. Fetuin-A may have the capacity to modulate inflammation, possibly benefiting the development of hypertension and its complications to some extent.

In general, based on the results above, fetuin-A could play a significant role in vascular health and may have a role in the pathogenesis of hypertension. More research is required to decode all aspects related to the clinical applications of fetuin-A, especially in relation to hypertension [23,24]. With increasing knowledge, the significant interactions between COVID-19 and cardiovascular diseases merit a more comprehensive study of the implications for this cohort in the convalescent phase [25,26].

## Materials and methods

Sample collection

Blood samples were collected from 115 patients (79 males and 36 females) who attended the Nasiriyah Heart Center in Dhi Qar Governorate, Iraq, between March 16, 2023, and August 25, 2023. The ages of the patients ranged from 5 to 93 years. All participants had experienced cardiovascular conditions following their recovery from COVID-19, and all had previously tested positive for and recovered from the virus. Individuals who had not been infected with COVID-19 were excluded from the study. The clotted serum samples were centrifuged at room temperature for 20 minutes at 2000-3000 RPM to separate the supernatant from the sediment. The samples were left to rest without shaking for no more than 10-20 minutes. The clear supernatant was carefully aspirated into a clean, labeled, screw-capped plastic vial and stored at -20°C, avoiding disturbance of the sediment. Testing had to be performed within one month to prevent the detrimental effects of multiple thawing cycles. The samples were allowed to temper at room temperature before assay to ensure precise readings. General laboratory safety measures were followed for each specimen collected and prepared.

Assay principle

The enzyme-linked immunosorbent assay (ELISA) kit used was provided by BT-Laboratory (Shanghai, China). The plate was pre-coated with human fetuin-A antibody. Fetuin-A in the sample bound to the antibodies on the wells. Biotinylated human fetuin-A antibody was then added, which bound to fetuin-A in the sample. Next, Streptavidin-HRP (Binder, Germany) was added, which bound to the biotinylated fetuin-A antibody. After incubating at 25°C for 10 minutes, unbound Streptavidin-HRP was washed away. A substrate solution was then added, and color developed in proportion to the amount of human fetuin-A present. The reaction was terminated by adding acidic stop solution, and absorbance was measured at 450 nm.

Method

During the entire process, all reagents, standard solutions provided by BT-Laboratory, and samples were prepared according to the manufacturer’s instructions, and all reagents were brought to room temperature before use. The assay was conducted at room temperature. The number of strips (JSHD, China) needed for the assay was noted, and the strips were inserted into frames for use, while the unused ones were stored at 2-8°C. Then, 50 μL of the standard solution was added to the standard wells, and 40 μL of the sample was added to the sample wells. The appropriate reagents were also added to the standard wells (excluding the blank control well). The plate was gently agitated to ensure thorough mixing, covered with a sealer, and incubated for 60 minutes at 37°C. After incubation, the sealer was removed, and the plate was washed five times with wash buffer by soaking the wells with 300 μL of wash buffer for 30 seconds to 1 minute for each wash. For automated washing, each well was aspirated or decanted and washed five times with wash buffer, followed by blotting the plate onto paper towels. Next, 50 μL of substrate solution A was added to the wells, followed by 50 μL of substrate solution B. The plate was resealed and incubated for 10 minutes at 37°C in the dark. The color changed immediately from blue to yellow upon the addition of 50 μL of stop solution to each well. The optical density of each well was determined immediately with a microplate reader (Shimadzu, Japan) set to 450 nm within 10 minutes after adding the stop solution.

Statistical analysis

Data analysis was performed using the Pandas library (Version: 1.3.3, Python Software Foundation, Beaverton, Oregon) and the SciPy library (Version: 1.7.1, NumFOCUS, Austin, Texas) in Python (Version: 3.8, Python Software Foundation, Beaverton, Oregon), which provide robust tools for data manipulation and statistical analysis. Python is one of the most flexible scientific languages with extensive statistical capabilities, and it was used primarily for this analysis. According to the central limit theorem, the distribution of the sample mean will be normal if the sample size is equal to or greater than 30. Therefore, normality of the samples did not need to be tested in this study, and there were no violations of the normality assumption. Categorical variables were described using proportions and frequencies, while continuous variables were summarized using mean and standard deviation (SD). A series of independent sample t-tests were performed to compare whether there were statistically significant differences between means. This test was chosen because it assesses the null hypothesis that the means of two independent groups are equal. In this study, p-values were calculated to determine whether the differences between groups were statistically significant. A significance level of p < 0.05 was used throughout the study.

## Results

Fetuin-A

**Figure 1:**
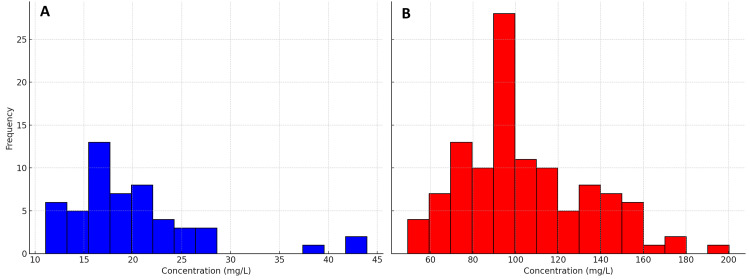
Concentration Level Distribution in Healthy Controls (A) and Patients (B)

Figure 1 displays a clear distinction in fetuin-A concentration levels between healthy controls and patients. The blue bars represent the concentration levels of the healthy control group, where fetuin-A concentrations are generally low, predominantly ranging from 10 to 30 mg/L, with a peak around 15-20 mg/L. In contrast, the red bars represent the concentration levels of the patient group, showing higher values predominantly between 60 and 140 mg/L, with a peak around 100 mg/L.

This suggests that lower fetuin-A levels are characteristic of healthy individuals, while the patient group exhibits significantly higher concentrations, mostly between 60 and 140 mg/L, with a notable peak around 100 mg/L. The wider range and elevated levels in patients suggest that increased fetuin-A concentrations may be associated with the health condition under study.

Table 1 shows that the mean concentration for patients is 103.64 mg/L, with a standard deviation of ± 30.96, while for the control group, it is 19.199 mg/L, with a standard deviation of ± 5.57. The p-value of 0.000 indicates a highly significant difference between the two groups. This suggests that there is a statistically significant difference in fetuin-A concentration between patients and the control group in this study.

**Table 1:** Mean Concentrations of the Measured Substance in Patient vs. Control Groups P<0.05 is considered statistically significant.

Group	N	Mean concentration (mg/L)	p-value
Patients	115	103.64 ± 30.96	0.000
Control	50	19.199 ± 5.57	

High blood pressure factor

**Figure 2:**
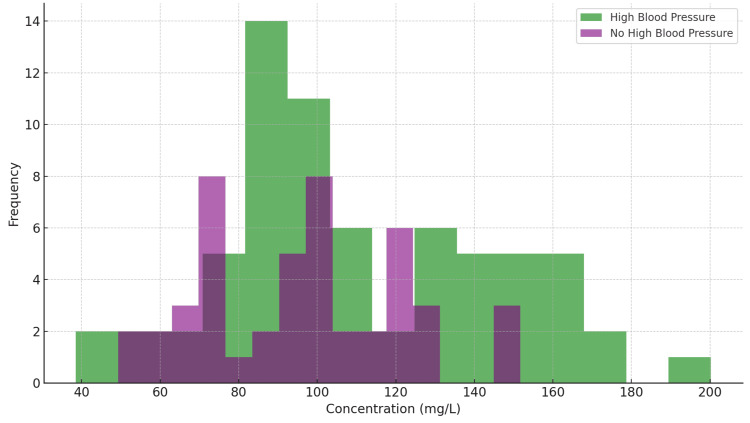
The Concentration Levels of Fetuin-A Against the High Blood Pressure Status

Figure 2 illustrates the distribution of fetuin-A concentration levels (measured in mg/L) among individuals with and without high blood pressure.

In the high blood pressure group (green bars), the fetuin-A concentrations tend to cluster around 80-100 mg/L and 120-140 mg/L. This indicates that fetuin-A levels in high blood pressure patients are relatively high, with the most prevalent concentration being about 100 mg/L. There is considerable variation in the higher concentration values, reaching up to 200 mg/L, indicating some variability in the data. On the other hand, the normal blood pressure group (purple bars) shows a more spread-out concentration distribution, generally lower than in the high blood pressure group. The highest frequency of concentrations is between 60 and 100 mg/L, with a second smaller peak around 120 mg/L. This suggests that individuals without hypertension tend to have lower fetuin-A levels compared to those with high blood pressure. The histogram clearly distinguishes between the two populations: those with high blood pressure exhibit higher frequencies of elevated fetuin-A concentrations compared to their counterparts. This trend may suggest a relationship between higher fetuin-A levels and high blood pressure.

Table 2 shows that among individuals with high blood pressure, the mean concentration is 109.01 mg/L with a standard deviation of ± 33.43, whereas for those without high blood pressure, the mean concentration is 95.88 mg/L with a standard deviation of ± 25.38. The p-value of 0.025 indicates a significant difference between these two groups. Therefore, high blood pressure appears to significantly influence the concentration of fetuin-A in this study.

**Table 2:** A comparison of Mean Concentrations Based on High Blood Pressure Status P<0.05 is considered statistically significant.

High blood pressure	N	Mean concentration (mg/L)	p-value
Yes	68	109.01 ± 33.43	0.025
No	47	95.88 ± 25.38	

## Discussion

The relationship between COVID-19 and cardiovascular diseases

This study further confirms the significant interplay between COVID-19 and cardiovascular diseases, a relationship increasingly recognized in recent research. Earlier studies have substantiated that the virus can have long-term adverse effects on the heart health of those infected [27-30]. The wide age range of patients in our study highlights an important observation: recovery from COVID-19 may be accompanied by persistent changes in cardiovascular biomarkers, including fetuin-A. This protein, known for its role in calcification and inflammation [20,21], is critical for understanding the long-term impact of the virus on cardiac health. These findings align with previous data.

Blood pressure and fetuin-A concentration

The study also identified a significant association between high blood pressure and elevated levels of fetuin-A, adding complexity to the existing literature. Our research recorded significantly higher levels of fetuin-A in individuals with high blood pressure, which contrasts with other studies that reported a decrease in fetuin-A levels as blood pressure increased [23]. This discrepancy may be due to the long-term effects of COVID-19, potentially altering the typical relationships between cardiovascular risk factors and biomarkers. The findings suggest that physiological changes induced by COVID-19 might affect fetuin-A levels differently compared to those without a history of COVID-19. Further research is clearly warranted to elucidate these relationships and the underlying mechanisms [5].

While these findings are insightful, the focus on patients at the Nasiriyah Heart Center and the sample size may limit the generalizability of the results to broader populations. Furthermore, the study's reliance on single-time-point measurements of fetuin-A and blood pressure is a notable limitation. Longitudinal data would offer a more comprehensive understanding of the fluctuations and long-term trends in these variables, which could provide deeper insights into their relationship with he health condition being studied, which may not fully capture the dynamic nature of these biomarkers over time. Longitudinal studies with repeated measures are necessary to confirm whether the observed changes in fetuin-A levels are sustained or fluctuate during the recovery process.

Future studies should aim to include larger, more diverse populations and account for a broader range of factors, such as age, sex, comorbidities, and pre-existing cardiovascular conditions. This would offer a more comprehensive understanding of the interplay between COVID-19 recovery and cardiovascular health.

## Conclusions

This supports the complex, multifactorial relationship between recovery from COVID-19 and cardiovascular health. The results showed that COVID-19 may exacerbate cardiovascular risk through marked elevations in biomarkers, including fetuin-A. Elevated levels of fetuin-A were observed in patients with high blood pressure, suggesting that COVID-19 likely alters the typical association between blood pressure and related biomarkers. This implies that post-COVID-19 patients may require more intensive monitoring and management strategies tailored to long-term cardiovascular health. Further research is necessary to understand the mechanisms behind these changes and to develop clinical guidelines that can more effectively manage cardiovascular health among COVID-19 survivors.
